# Anesthetic Management of Iatrogenic Tracheal Injury: A Case Report

**DOI:** 10.7759/cureus.58158

**Published:** 2024-04-12

**Authors:** Afnan Amjad, Faraz Mansoor, Fattahullah Khan, Shehzad Khan, Aysha Mairaj

**Affiliations:** 1 Anesthesia and Critical Care, Shaukat Khanum Memorial Cancer Hospital and Research Centre, Peshawar, PAK; 2 Internal Medicine, Shaukat Khanum Memorial Cancer Hospital and Research Centre, Peshawar, PAK

**Keywords:** emergency, anesthesia, esophagectomy, double lumen tube, tracheal perforation

## Abstract

Tracheal perforation following oesophagectomy is a very rare and occasionally life-threatening condition that requires a high degree of suspicion and early intervention for optimal patient outcomes. This article presents a case report of a 46-year-old male who presented with respiratory failure secondary to tracheal perforation at the level of carina following a two-stage oesophagectomy. He underwent a second emergency procedure; the airway was secured with a left-sided double-lumen tube, and tracheal perforation was successfully repaired. This case report will briefly cover the challenges and difficulties faced by anesthetists in the airway management, ventilation, and hemodynamic instability of such patients.

## Introduction

Tracheobronchial injury is a very uncommon condition, which is associated with significant morbidity and mortality. While head and neck, and chest trauma resulting from motor vehicle accidents is considered the most common cause, it can occasionally result from endotracheal intubation, tracheostomy, head and neck, and oesophageal surgeries, rigid bronchoscopy, metallic airway stent insertion and removal, and tumor invasion [1-2]. Clinical features of tracheal perforation in alert patients include sub-cutaneous emphysema (35% to 85%), pneumothorax (20% to 50%), haemoptysis, and neck or chest pain. Some patients with small injuries may be asymptomatic. Intubated patients with tracheal injuries might pose challenges in mechanical ventilation because of the air leak. Pneumomediastinum and pneumothorax are commonly observed radiological signs. Computed tomography and bronchoscopy are required to confirm the diagnosis and make a treatment plan. Management of tracheal injuries includes conservative management, stent placement, open operative repair, and endoluminal repair. Different ventilatory strategies can be used for the repair of tracheobronchial injuries including spontaneous ventilation, high frequency jet ventilation, cardiopulmonary bypass, employing a double lumen tube and standard orotracheal intubation [3].

## Case presentation

A 46-year-old male (height 185 cm; weight 93 kgs) previously diagnosed with type-2 diabetes presented to the emergency department with breathing difficulty, cough, and fever persisting for three days. Notably, the patient had undergone a Hybrid 2-stage Ivor Lewis two-stage oesophagectomy procedure 10 days back for underlying esophageal squamous cell carcinoma. The following vital signs were observed on admission: heart rate 110 per minute, blood pressure (BP) 140/100 mmHg, respiratory rate 23-30 per minute and oxygen saturation 84% on room air. On physical examination, he was fully alert and oriented, chest auscultation revealed decreased air entry on the right side and bilateral crepitations. Arterial blood gas analysis showed a pH of 7.51, partial pressure of oxygen (PaO2) of 48 mmHg, and partial pressure of carbon dioxide (PaCO2) of 33 mmHg. A bedside chest X-ray was requested which showed patchy consolidation on the right side of the chest suggestive of an underlying infective etiology. In the context of the recent history of surgery and underlying malignancy, an emergency computed tomography pulmonary angiogram (CTPA) with oral contrast was performed which revealed a spectrum of findings, including dehiscence at the anastomotic site in the lower esophageal region, fistulous communication between the trachea and the neo-esophagus at the carina level, oral contrast pooling in the right pleural cavity and distal trachea, a large right-sided pneumothorax leading to the collapse of the right middle and lower lobes, and the presence of subcutaneous emphysema along the right lateral thoracic wall extending into the back region, accompanied by a defect in the superficial muscles.

**Figure 1:**
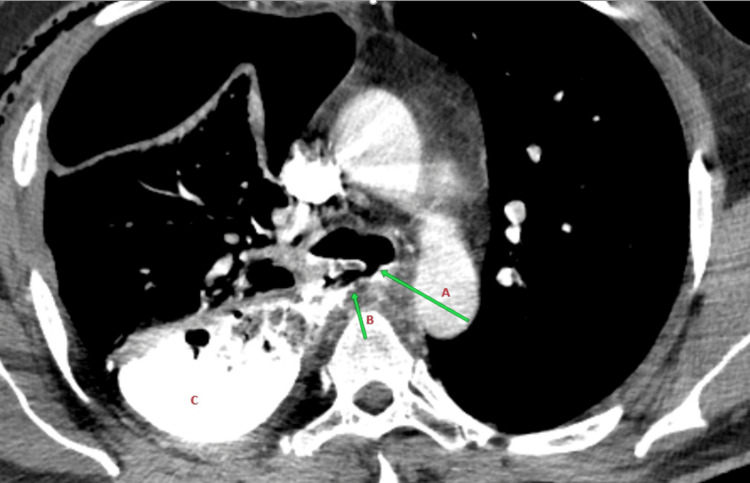
Computed tomography (CT) with contrast after tracheal perforation (A) Tracheal Perforation just above Carina (B) Oesophageal Perforation (C) Contrast Pooling in the Right Pleural Cavity

An emergency thoracotomy was planned, and the patient was shifted to the preoperative holding area. Necessary paperwork, including a high-risk consent, was completed. The patient was attached to the bedside cardiac monitor. On examination in the holding area, his temperature was found to be 36.7°C with a heart rate of 91 beats/min and respiratory rate of 22 breaths/min, and he required supplemental oxygen of 10 L/min to maintain an oxygen saturation of more than 90%. The WHO surgical safety checklist was completed as part of the preoperative preparation. Following completion of the checklist, the airway was secured with a left-sided 37-size double-lumen tube, and its position was confirmed with the help of the fiberoptic bronchoscope. A radial arterial line was inserted into the right radial artery to facilitate continuous blood pressure monitoring. Isoflurane was used for anesthesia maintenance, complemented by atracurium for muscle relaxation. The operative course was uneventful, and at the end of the procedure, the double-lumen tube was switched to a single-lumen tube. However, the patient had a very stormy post-operative course, including difficulties in oxygenation and ventilation, and worsening of the chest X-ray. A thoracic epidural was placed for post-operative pain control before shifting the patient to the intensive care unit.

## Discussion

While trauma to the head and neck, and chest is the most common cause of tracheal injury, iatrogenic tracheal injuries are rare yet serious complications in clinical practice. Existing literature on this subject is limited, highlighting the low incidence of such occurrences. Notably, the incidence of tracheal tears during transhiatal esophagectomy is reported to be less than one percent and it can pose challenges for the anesthesiologists [4]. In addition, tracheal injury may also result as a consequence of endotracheal intubation. Immediate clinical sequelae following tracheal tears include subcutaneous emphysema, pneumothorax, pneumo-mediastinum, tracheobronchial hemorrhage, and difficulties during positive pressure ventilation. These patients may require prolonged intensive care unit stay with ventilatory support. Given these complexities, anesthesiologists, emergency physicians, and intensivists must be well-versed in its clinical presentation, management of life-threatening complications, intra-operative skills focusing on airway management, and post-operative care for these patients in both the intensive care unit and the wards.

It is estimated that approximately 80% of the patients with tracheobronchial injuries resulting from blunt trauma succumb to death before reaching the hospital [5]. A tracheobronchial tear is more common in penetrating trauma than blunt trauma. Approximately 0.5 to 2% of blunt trauma patients admitted to the emergency department have underlying tracheobronchial injury [6]. The presence of subcutaneous emphysema, pneumothorax and pneumomediastunum should raise the index of suspicion of tracheobronchial injury in these patients. Computerized tomography and fibreoptic bronchoscopy stand as the preferred investigations. Securing the airway is an important part of the emergency management of these patients and it is recommended by the American Society of Anesthesiologists to maintain spontaneous breathing until the airway is secured [7]. A tracheostomy tube can be inserted through the wound in cases of complete transection of the proximal trachea where orotracheal intubation is not possible. Unfortunately, most of the tracheal injuries remain undetected at the time of their presentation. Incorrect positioning of the endotracheal tube on chest X-ray, for example, beyond the tracheal anatomy, and persistent air leak after placement of intercostal tube have high sensitivity and specificity [6]. Considering the multifaceted nature of tracheobronchial injury, it is important for the healthcare professionals to look at the intra-operative and post-operative management strategies.

The exact incidence of tracheobronchial injuries related to endotracheal intubation is unknown. It is believed that endotracheal intubation is the most common cause of tracheobronchial injury. As per Schneider et al., the reported incidence rates are one in 75000 for standard tube intubation, 5-19 per 10,000 for double lumen tubes, and one in 575 for percutaneous tracheostomies [8]. During the intra-operative phase, the tracheobronchial injury should be suspected in the presence of subcutaneous emphysema, pneumothorax, high airway pressure, and sudden fall in Tidal volume resulting in inadequacy of gas exchange. Despite the fact that a left-sided double-lumen tube was used in the case described here, it is highly unlikely that the tracheal injury was caused by the intubation as there was a communication between the anastomotic site of the oesophagus and stomach with the trachea at the time of the diagnosis. Most of the tracheal tears are managed surgically and those close to the carina require single lung ventilation.

A double-lumen tube is usually the preferred choice for single-lung ventilation. A left-sided DLT was employed in this case because of the surgical requirements for right thoracotomy. In addition to the double-lumen tube, various other ventilator strategies are described in the literature. For example, cardiopulmonary bypass, extracorporeal membrane oxygenation and standard orotracheal intubation [9]. These patients can be safely extubated at the end of the surgery provided there is no other indication for continuing the ventilator support in the intensive care unit. A thoracic epidural is effective for post-operative pain management.

## Conclusions

In conclusion, tracheal injuries, though rare, present significant challenges to clinicians across multiple specialties. While trauma remains a primary cause, iatrogenic injuries, particularly during procedures like transhiatal esophagectomy and endotracheal intubation, underscore the importance of vigilance and expertise in managing these complex cases. The diverse clinical presentations, ranging from subcutaneous emphysema to pneumothorax, demand a thorough understanding of diagnostic modalities such as CT scans and bronchoscopy. Prompt recognition and intervention are vital, with a focus on securing the airway and addressing life-threatening complications. Effective post-operative pain management, such as thoracic epidurals, complements the comprehensive approach necessary for these patients.

## References

[1] Miñambres E, Burón J, Ballesteros MA, Llorca J, Muñoz P, González-Castro A (2009). Tracheal rupture after endotracheal intubation: a literature systematic review. Eur J Cardiothorac Surg.

[2] Kalverkamp S, Störmann P, Graeff P, Raab S (2023). Traumatic tracheobronchial injuries - recommendation of the interdisciplinary working group of the DGT and DGU to establish a uniform classification for diagnostics and therapy (Article in German). Zentralbl Chir.

[3] Dereeper E, Karler C, Roman A, Cadière GB, Urbain F, Sosnowski M (2005). The anesthetic management of a case of tracheal necrosis. Anesth Analg.

[4] Munasinghe BM, Karunatileke CT (2023). Management of an intraoperative tracheal injury during a Mckeown oesophagectomy: a case report. Int J Surg Case Rep.

[5] Ecker RR, Libertini RV, Rea WJ, Sugg WL, Webb WR (1971). Injuries of the trachea and bronchi. Ann Thorac Surg.

[6] Karmy-Jones R, Wood DE (2007). Traumatic injury to the trachea and bronchus. Thorac Surg Clin.

[7] Ajith AK, Anjum F (2023). Tracheobronchial tear. https://www.ncbi.nlm.nih.gov/books/NBK560900/.

[8] Passera E, Orlandi R, Calderoni M (2023). Post-intubation iatrogenic tracheobronchial injuries: the state of art. Front Surg.

[9] Al-Thani H, Ahmed K, Rizoli S, Chughtai T, Fawzy I, El-Menyar A (2021). Utility of extracorporeal membrane oxygenation (ECMO) in the management of traumatic tracheobronchial injuries: case series. J Surg Case Rep.

